# Women's Health Surveillance: Implications for Policy

**DOI:** 10.1186/1472-6874-4-S1-S31

**Published:** 2004-08-25

**Authors:** Sari Tudiver, Mireille Kantiebo, Jean Kammermayer, Monica Mavrak

**Affiliations:** 1 Women's Health Bureau, Health Canada, Tunney's Pasture, Ottawa, K1A OK9, Canada; 2 Women's Health Bureau, Health Canada, Tunney's Pasture, Ottawa, K1A OK9, Canada; 3 Women's Health Bureau, Health Canada, Tunney's Pasture, Ottawa, K1A OK9, Canada; 4 Women's Health Bureau, Health Canada, Tunney's Pasture, Ottawa, K1A OK9, Canada

## Introduction

The previous articles in this supplement provide valuable data and insights about women's health in Canada but also point to significant gaps in information gathering about women's health and about gender differences in health. These gaps are evident in health surveillance activities and in areas of biomedical and social research. As well, the gender implications of social and economic policies are rarely considered in a systematic and consistent way. This long-standing situation is the result of assumptions and values underlying theoretical and practical approaches to data collection, research, analysis and policy development, which have tended to reinforce the centrality of women's reproductive and caregiving roles and ignore or underplay women's experiences in other sectors of social life. [1-4]

Identifying and redressing the sex/gender gaps in health surveillance would contribute to a more robust and accurate system of health surveillance in Canada and, in turn, provide a stronger evidence base for the development and implementation of effective social policies to improve population health outcomes and reduce health inequalities. More effective policies could lead to the identification of new areas and methods for improved surveillance. This could be achieved, in part, by systematically incorporating gender-based analysis into surveillance practices, particularly by focusing on the context and diversity of people's lives; developing and applying gender-sensitive health indicators; and using innovative theoretical concepts and analytic tools to map the pathways and interrelations between population health determinants and health outcomes. This chapter underlines the need for a policy framework for women's health surveillance in Canada and points to some of the elements that such a framework might include.

## Surveillance and the Policy Cycle

Surveillance data contribute to policy development in a number of ways. Surveillance is used to identify sentinel events, such as outbreaks of diseases, that may require immediate policy decisions concerning public health. Surveillance systems track the incidence of particular diseases, such as diabetes, breast cancer and sexually transmitted infections, over time, as well as rates and patterns of health behaviours, such as smoking, to which policies and programs are directed. Data may show particular subgroups at increased or decreased risk of these and other health conditions – important information for health promotion programs, research and clinical treatment.[1,5] Societal trends identified through surveillance – for example, the rising age of women at first birth[6] – can be further analyzed to identify correlates such as level of education, income and employment; increased use of technologies for assisted human reproduction; attitudes towards child-rearing; and the possible causes of the trend. Such analysis is essential to the development of healthy public policy.[7]

The various stages through which a policy must pass to be approved and implemented are often referred to as the "policy cycle," or policy process.[8,9] These stages commonly involve identifying the issue; gathering available research and other forms of evidence; conducting risk assessments; consulting with stakeholders; developing and refining policy options; making decisions at the appropriate levels of government; implementing policy; and assessing its impacts over time.[10] Ideally, high-quality evidence and analysis are accessible to those involved at all stages of the policy cycle. Health surveillance data are especially relevant to the detailed and systematic delineation and analysis of an issue, including risk assessment. Gaps in surveillance data may identify the need for additional research before or during the development of policy options. When urgent policy decisions must be taken, the phases of the policy cycle may be carried out simultaneously, in a rapidly changing environment.

Surveillance is also pertinent to the implementation and assessment of policies. Through the use of appropriate health indicators (see "Developing Women's Health Indicators" below), surveillance can be part of a monitoring system to track the effectiveness of specific interventions, such as health education or screening programs, and of broad policies, such as health system restructuring, in improving population health outcomes and reducing health inequalities.

A number of factors limit the usefulness of current surveillance data to the policy process. Many of these have been identified in previous chapters. To summarize, there has been no comprehensive monitoring and reporting on women's health. Where data are gathered, they reveal methodological constraints: the National Population Health Survey (NPHS) and other surveys rely on memory through self-reporting (see chapter entitled "Body Weight and Body Image"); there are data inconsistencies between survey years; and cross-sectional surveys do not provide causal explanations. The methods used may lead to under-reporting, making the data less robust for understanding the situations of particular subgroups. This is the case in surveys on violence that do not include people without telephones, those who are homeless or in institutions, or those in the Yukon, Northwest Territories and Nunavut, many of whom are at high risk of maltreatment (see chapter entitled "Violence against Canadian Women"). Detailed health surveillance information pertaining to Aboriginal and other ethno-cultural communities, people with disabilities, and residents of rural and remote areas is also sparse, limiting an understanding of sex and gender differences in these populations (see the chapter entitled "The Mortality, Health and Life Expectancy of Canadian Women"). There is a lack of infrastructure for standardized reporting on many public health issues and a need for cultural and social sensitivity when such data are gathered. There is also a lack of co-operation on the standardization of surveillance among jurisdictions (see chapter entitled "Gender Differences in Bacterial STIs in Canada"). Some of these problems are the result of limited resources.[11]

## Broadening the Scope of Surveillance and Analysis

The design of surveillance systems can be limited when existing policies assume a focus that is too narrow, such that important aspects or consequences of policy interventions are missed. To be relevant to policy, surveillance must be designed to capture a range of data about the context of health behaviours and the interplay between the social and biological determinants of health, including sex and gender differences. Gender-based analysis offers a systematic, analytic tool that can be used to examine diversity within and between populations and subgroups (according to age, socio-economic status, sexual orientation, race, ethnicity, education, abilities, location, etc.) and across the life cycle.[12,13] Sex and gender are more than independent variables, since exploring these differences often challenges the assumptions underlying analytic frameworks, including interpretation of behaviours, and points to the need for different levels and types of data collection, analysis and intervention.[14]

Health system reform provides an example of how the framework used for data gathering must be able to explore complex causal pathways and anticipate possible future effects of policies. For example, data on shortened hospital stays have been used to track cost savings and patient health outcomes. However, the impact of early discharges on unpaid family caregivers, the majority of whom are women, has not traditionally received attention as a significant issue for surveillance and policy.[15,16] There is growing evidence that caregiving increases the risks of morbidity and mortality[17,18]; this is of particular relevance to mid-life and older women, who may have chronic conditions such as arthritis or diabetes. Research into the economic, social, physical and mental health effects of added caregiving could provide a basis for the development of health indicators with which to measure the impacts of health system changes on the health and well-being of caregivers, both women and men.

## Developing Women's Health Indicators

Health surveillance systems report on health indicators, defined as statistics or parameters that provide, over time, information on trends and changes in the condition and status of health.[19] Health indicators are important tools that help describe and measure the determinants of health, including health services, as well as health status and health outcomes. They are useful for formulating policies, programs and legislation, and are used to monitor and report on progress towards health goals and objectives. Indicators can inform health impact assessments, and social and financial costing. Indicators also permit comparisons between jurisdictions against established standards.

Traditional health indicators, based exclusively on sex-disaggregated data, do not adequately reflect the interrelations between biological processes, social roles, socio-economic context, the health care system and health outcomes. Various types of statistical analysis, such as multivariate analysis, incorporate some considerations of social roles and other aspects of gender, but the challenge is to develop indicators that reflect the complex interconnections among health determinants and health outcomes, including key differences in health and well-being between women and men, boys and girls. [20-22]

For instance, the chapter in this report on "Multiple Roles and Women's Mental Health in Canada" demonstrates how single employed and unemployed mothers have high rates of personal and chronic stress. This suggests a need to determine how cumulative stress levels contribute to chronic diseases or other health conditions for women and men. To track sex and gender differences in the occurrence of chronic diseases or other health problems, indicators should capture the interaction between biological, socio-economic and behavioural factors, cumulative exposures to different types of stress (e.g. in workplaces, in families), and patterns of health problems, such as heart disease, among women and men. Further, research showing an association between infant and childhood risk factors and adult chronic conditions, including heart disease, points to the need for indicators to reflect the multiplicity of interactions across the lifespan.[23,24] Emerging theories in social epidemiology offer important constructs to explore the "cumulative interplay between exposure, susceptibility and resistance."[25] Such theories are based, in part, on increased understanding of the interrelations between the psychological and the somatic, especially the impacts that stressors, such as discrimination and early deprivation, have on human health.

If social policies are to *promote *health as well as *prevent *disease, indicators must be designed to identify a broad range of human behaviours and the conditions and context that shape behaviours. Researchers within Aboriginal communities suggest that, in addition to focusing on patterns of disease and consequences of victimization, indicators for Aboriginal health should be constructed to capture health-seeking behaviours that reflect positive coping strategies and the resilience of individuals and communities. [26-28] Similar perspectives have been articulated by researchers from immigrant and refugee communities and disability rights organizations, among others. [29-31] Earlier in this report, Wong et al. demonstrate that standard indicators on the sexual health of Canadian adolescents are constructed to identify diseases (e.g. sexually transmitted infections) and negative outcomes (e.g. unplanned pregnancies), with little attention to indicators of behaviours and healthy sexuality. As they note, indicators need to represent "a broad-based behavioural, biological and cognitive approach to adolescent sexual health" (see chapter entitled "Sexual Health").

There are limits to structured surveillance tools, including well-defined indicators. Surveys and indicators must be augmented and informed by qualitative research to reveal the context behind the limited answers available through traditional indicators. Other sources must be critically mined for evidence on sex, gender and diversity to answer policy-relevant questions: "Why did this trend or pattern occur?" "What are the short- and long-term implications for the health of women and men and for particular subgroups?" "What specific policies and interventions are likely to be most effective in achieving improved health outcomes and reducing health inequalities?" A gender lens can be applied to historical reviews of trends and policies, other theoretical and analytic work, biomedical and social research, policy research and evaluation, risk assessments, environmental scans and health technology assessments to achieve a more comprehensive understanding of an issue and to further refine indicators for women's health surveillance.

## Developing Gender-Sensitive Policies

Earlier chapters offer a number of recommendations for further areas of surveillance, research and analysis on women's health. They also identify the need for specific social policies and programs to be undertaken by appropriate levels of government, health professions and other non-governmental organizations to improve health outcomes and reduce health inequalities. Some issues, such as sexual and reproductive health, smoking, cardiovascular disease and family violence, have a range of surveillance data, research and policy associated with them that could form the basis for comprehensive, gender-sensitive social policy initiatives. Two of these issues, sexual and reproductive health, and smoking, will be discussed here to briefly illustrate how such policies might emerge.

### Sexual and Reproductive Health

A broad social policy initiative is needed to address the sexual and reproductive health of females and males across the life cycle. Issues include the prevalence of sexually transmitted infections (chlamydia, human papillomavirus and HIV) among young and older women; social and economic factors that limit women's capacity to negotiate safer sex; and lack of information about, or access to, birth control.[32] Canadian males share concerns about STIs and sexual dysfunction. Male-related causes of infertility have also received attention, because of increasing evidence of possible links between decreased male fertility and exposure to pesticides or other toxicants. However, the application of technologies for assisted human reproduction tends to focus on women.

As the relevant chapters in this report show, there exist sex-disaggregated surveillance data and other sources of evidence pertinent to sexual and reproductive health in Canada, but there are gaps in the integration of data across jurisdictions. There is also considerable biomedical and social marketing research on contraceptive methods and on the promotion of healthy sexuality and sex education, much of which focuses on male and female adolescents and young adults, with less emphasis on other age groups.

Framework documents developed through consultations with federal and provincial/territorial governments and the Canadian public clarify values and articulate ethical guidelines and approaches to these sometimes controversial issues.[33] As well, Canada is signatory to a number of international agreements that include commitments to improve maternal health, promote sexual and reproductive health and rights, ensure the availability of universal access to reproductive health services, and promote gender equality and women's empowerment. [34-36]

Ideally, the development of an integrated, gender-sensitive policy initiative for sexual and reproductive health would be part of a broad, inter-sectoral framework based on evidence that demonstrates the benefits for sexual and reproductive health outcomes of economic security, good nutrition, family life education, quality reproductive health services and empowerment. The framework would recognize that women and men of differing ages, socio-economic status, geographic locations, ethno-cultural backgrounds, abilities, and sexual orientations have different concerns and needs, and differ in access to resources, including health services. An integrated policy would be based on the effectiveness of strategies for improving sexual and reproductive health. Policies and programs could support access to effective birth control methods by both partners and programs that encourage self-esteem and skills to negotiate safer sex practices and respond effectively to situations of maltreatment/violence and power differentials.

Policies and programs could address the needs of diverse groups, including vulnerable populations of women and men, at particular stages in the life cycle. For example, gay and lesbian youth are at increased risk of mental health problems and would benefit from peer support or other programs. People with disabilities have identified the need for education and other programs related to sexual and reproductive health and choices. Involving those most directly affected in the various stages of the policy cycle, including the design of policy and programs, is associated with more successful outcomes.

Implementing a comprehensive policy on sexual and reproductive health requires alignment of relevant policies and programs already in place; development and application of health indicators that include positive aspects of sexuality for males and females from infancy to the older years; enhanced integration of surveillance systems that gather relevant data from different levels of government; identification of gaps and coordination of needed research, including policy research; and assessment of services, programs and policies. Databases of best practices and evaluations of interventions in Canada and internationally would be a highly useful resource for citizens, professionals, front-line workers and policy makers in the development of effective policy and in finding the "right mix" of interventions.[7]

### Smoking

Smoking is a modifiable risk factor for many diseases and for premature mortality. As the chapter in this volume entitled "Sex and Gender Differences in Smoking and Self-Reported Indicators of Health in Canadian Women" indicates, considerable evidence documents the numerous and serious health effects of smoking on both females and males, including increased risk of lung cancer and cardiovascular disease. Some effects of smoking are unique to women's physiology and life cycle. For example, women smokers have higher rates of cervical cancer and more menstrual problems, and they tend to experience menopause up to two years earlier than non-smokers. Smoking during pregnancy is associated with lower infant birth weights and other complications.

Existing surveys such as the Canadian Tobacco Use Monitoring Survey (CTUMS) and the NPHS show variations in rates and trends in smoking between males and females and among specific subpopulations of women in Canada. In general, smoking rates in all age groups, including teenaged girls and young women, have been decreasing since 1985. [37-40] However, smoking is an issue of particular concern for young females. It has been observed that girls begin smoking at earlier ages than boys, following the pattern of their earlier maturation. As well, the various surveys of smoking behaviour show that a greater percentage of girls aged 15 to 17 consistently report being current smokers than their male counterparts (although by age 18 to 19, teenaged boys generally either catch up to or surpass them). Early smoking carries particular health risks for females.[41] There are long-term implications for population health and for costs to the health care system if teens who currently smoke continue to do so into adulthood.

Smoking is an indicator of social and health inequality, and reveals a clear socio-economic gradient. Smoking is more prevalent among women in low-income households, women who have low-status jobs, are single parents or divorced, and those with low levels of education (see "Sex and Gender Differences in Smoking and Self-Reported Indicators of Health in Canadian Women"). Women tend to smoke for somewhat different reasons than men: as a coping strategy for feelings of stress and lack of control over their lives, as part of a daily routine to take a break from caregiving and other work, as time to share intimacies with partners or friends, or to "distance and defuse relationships" and control negative emotions. Images of smoking as "cool" and a way to ward off weight gain have influenced many female teens and young girls who smoke.[42] Many older women face barriers to quitting, including fear of weight gain, lack of confidence, and lack of support to overcome this addiction.

The Federal Tobacco Control Strategy (FTCS) combines a variety of approaches to achieve measurable goals in reducing the prevalence of smoking in Canada, including a mass media campaign; protection, prevention, cessation and harm reduction initiatives; and taxation on tobacco.[43] Reviews of best practices pertaining to smoking cessation strategies for youth, pre- and post-natal mothers, and other target groups are being compiled and disseminated. On-line self-help programs and other resources are available.

The application of gender-based analysis to smoking issues and an understanding of the social and economic determinants of smoking provide the basis for a more gender-sensitive tobacco reduction policy in Canada. This approach has been articulated in *Filtered Policy: Women and Tobacco in Canada *(2000), which suggests the use of broad policy measures related to determinants of health, including income adequacy, child care and other areas of women's work, to reduce tobacco use among women and to avoid increasing social inequalities.[44] Policy initiatives pertaining to women and tobacco were also reinforced with the adoption of the Framework Convention for Tobacco at the World Health Assembly in May 2003, which called for measures to address gender-specific risks when developing tobacco control strategies.[45]

A serious addiction, smoking can be influenced by a combination of gender-sensitive social and economic policies and by targeted programs that address the diversity of individual and group barriers to reducing or quitting smoking. Surveillance and various forms of research, including policy research, are integral to the development of tobacco control policies and programs and to monitoring their effectiveness in improving health outcomes for men and women, girls and boys.

## From Surveillance to Policy Action – and Back

This report proposes a significant paradigm shift in the gathering of health surveillance data in order to yield a more profound and accurate understanding of the determinants of women's health and health behaviours. Such a shift is part of an interactive process in which surveillance informs the stages of policy development, implementation and evaluation, and the various stages of the policy cycle generate new questions and approaches to surveillance. The *Federal Plan for Gender Equality*,[46] Health Canada's *Gender-Based Analysis Policy*[12] and Health Canada's *Women's Health Strategy*[13] provide the mandate and policy guidelines for the consistent application of gender-based analysis to all relevant programs, policies, legislation, research and surveillance activities. Some further strategies for sustaining a dynamic process follow.

### Collaboration

Surveillance systems are costly and involve a variety of stakeholders within and across jurisdictions. As a result, competing priorities may pose obstacles to the gathering of new data. Improved collaboration across federal departments and among jurisdictions and sectors is crucial to ensuring that stakeholders understand the rationale for proposed changes and the value added to the work of others who will use the proposed data and analyses. Interdisciplinary work is challenging, in part because each expert comes with particular assumptions and a discourse that may be unfamiliar to others. Paradigms may be difficult to explain, but the collaboration of demographers, statisticians, social epidemiologists, policy analysts, qualitative researchers and gender experts on common projects can lead to creative synergy and innovative design of surveillance systems.

### Use of Evidence and Theory

There is a need for coherent theoretical frameworks that help to explain the dynamic interrelationships among the social and biological determinants of health, including processes of human resilience and vulnerabilities, causal pathways and cumulative effects of circumstances and risks over the life cycle. Further, there must be the analytic capacity and the commitment to use and refine the knowledge gained. Despite the need for sound evidence in the policy process and in clinical practice, research shows that the best available evidence is not always disseminated, considered or applied.[47]

For example, it is widely known that to achieve improved health outcomes and reduce health inequalities, governments must focus macro-level social and economic policies on poverty reduction, improved living and working conditions, and safer physical environments; strengthen communities and social networks ("social capital"); improve health system responses; and influence modifiable risk factors while remaining sensitive to the particular circumstances of people's lives, including differences in location. Yet, *individual *health behaviours and a concern with genetic determinants are often emphasized in research, health policies and therapies, and less consideration is given to social, economic and environmental determinants of health.[48]

### Policy Evaluations

To achieve effective social policies and to plan for the future, evaluation data on the impacts of current or past social policy initiatives are sorely needed. However, few countries engage in systematic health and social policy assessments. For example, the 1998 Acheson Report in the United Kingdom identified the increasing gap in inequalities in health, as did the Black Report of 1980, but did not assess the effects that social and economic policies implemented in the 1980s and 1990s may have had.[49]

The Netherlands provides a unique model, having undertaken systematic research over the past decade to map the nature and determinants of socio-economic inequalities in health and then to launch a program of intervention studies to compare health outcomes or process measures in experimental and control groups. A strategy was developed for reducing socio-economic inequalities in health, with specific recommendations and quantitative policy targets.[50]

This model has the potential to be adapted to policy research in other countries, including Canada. It would be enhanced by the application of gender-based analysis through all stages of the policy cycle.

### Public Involvement

A robust process, in which surveillance informs the policy process and policy guides surveillance, must incorporate authentic mechanisms for public involvement. Women's groups and organizations in Canada have a long and vibrant history of advocacy and engagement with federal, provincial and territorial governments in efforts to improve women's health. [51-53] Women of diverse ethno-cultural backgrounds, geographic locations and sexual orientations, and with different skills, education, abilities and disabilities have identified issues of concern, such as violence and poverty, and advocated to have these issues placed on the social policy agenda.

Some women's health groups have called attention to emerging international issues, such as the rapid development of reproductive and genetic technologies.[54] Women and Health Protection, a network of health providers, consumers and researchers, is engaged in research and informal surveillance on the impact of drugs and devices on women's health and provides input to government on policies pertinent to health protection.[55] Others have identified gaps in health planning, encouraged the integration of gender-based analysis into government processes and called attention to the need for further development of indicators to evaluate progress towards gender equality.[56] The National Coordinating Group on Health Care Reform and Women monitors the impact on women of Canadian health care system reforms, with a particular focus on home care.[57]

Such groups provide diverse perspectives, often "from the margins"; bring synergy and balance to discussions and debate; challenge assumptions and concepts; and suggest options to government for surveillance, research and policy. A wide range of women's voices can be heard through consultations, panels, advisory committees and working groups. Such input is vital to a transparent process of policy development. Successful implementation of effective health surveillance and social policies depends on a broad base of public dialogue and support.

## Conclusions

Surveillance data are subject to many limitations, including a lack of infrastructure for standardized reporting. There are also conceptual limitations to surveillance, particularly when data may be disaggregated by sex but provide no further evidence about gender differences.

Creative social policies can guide surveillance beyond these conceptual limits. To be relevant to policy development, an understanding of health determinants should be integrated into the framework of surveillance systems, to capture the diverse contexts of people's lives across the life cycle. Surveillance systems should also be designed to anticipate future trends and health information needs; for example, by monitoring the short- and long-term physical and mental health impacts of genetic testing and reproductive technologies, and the ways in which these may differ for women and men, boys and girls.

Surveillance systems can also be designed to monitor crosscutting issues relevant to many aspects of population health. Thus, surveillance data are crucial in occupational health because working conditions contribute to, or are a major cause of, chronic and other diseases and injuries experienced by women and men. Workplace conditions and exposures play a role in pulmonary conditions, cardiovascular disease, reproductive health, mental health issues and musculoskeletal illnesses, among others. Without detailed, gender-sensitive data on the conditions and structure of work over time and on the health of workers, these relations cannot be documented or addressed through workplace and other social policies.[2,58,59] Similarly, surveillance data on family violence contribute to a better understanding of a wide range of health issues from addictions to injuries to various somatic complaints (see the chapter on "Violence against Canadian Women").

Health surveillance systems should be able to alert governments and the public to social policy and program failures and contribute to analysis of the lessons learned. Carefully designed surveillance can be a "sentinel system" for the mix of innovative initiatives and policies that will improve population health outcomes, reduce economic and social inequalities, and enhance the quality of life for the most vulnerable in Canadian society.

## Notes

This report represents the views of the authors. It does not necessarily represent the views of the Canadian Population Health Initiative, the Canadian Institute for Health Information or Health Canada
